# Determinants of knowledge, attitudes, and practices in relation to HIV/AIDS and other STIs among people with disabilities in North-Shewa zone, Ethiopia

**DOI:** 10.1371/journal.pone.0241312

**Published:** 2020-10-27

**Authors:** Alemayehu Gonie Mekonnen, Alebachew Demelash Bayleyegn, Yared Asmare Aynalem, Tigist Demssew Adane, Mikyas Arega Muluneh, Abayneh Birlie Zeru

**Affiliations:** 1 Department of Nursing, College of Health Science, Debre Berhan University, Debre Berhan, Amhara Regional State, Ethiopia; 2 Department of Midwifery, College of Health Science, Debre Berhan University, Debre Berhan, Amhara Regional State, Ethiopia; 3 Department of Public Health, College of Health Sciences, Debre Berhan University, Debre Berhan, Amhara Regional State, Ethiopia; The Chinese University of Hong Kong, HONG KONG

## Abstract

**Introduction:**

People with disabilities face socioeconomic disadvantages and they have limited access to sexual and reproductive health information. They are highly vulnerable to sexual abuse which places them at increased risk of HIV and STI infection. At present, however, little is known about the knowledge, attitude and practice of sexually transmitted diseases including HIV/AIDS and other STIs in Ethiopia. This study aimed to identify which individual factors best predict knowledge, attitudes, and practices in relation to HIV/AIDS and other STIs among people with disabilities in North-shewa zone, Ethiopia.

**Methods:**

A cross-sectional study was conducted from June to October 2019. A total of 397 respondents were interviewed using a structured and pre-tested questionnaire. A systematic sampling technique was employed to select the respondents. Logistic regression was performed to analyze the data. A significant association was declared at a p-value of less than 0.05.

**Results:**

Nearly half of the study participants were knowledgeable in relation to HIV/AIDS (47.3%) and STIs (46.9%). Sixty-two percent of respondents had good attitude towards evidence of HIV/AIDS while sixty-nine percent of participants had good attitude towards helpful facts of STIs. Twenty-three percent of study participants had been ever tested for HIV infections. Being married (AOR = 2.23; 95% CI = 1.92, 10.72) was associated with having good knowledge of STI. Males were 1.6 times more knowledgeable about HIV/AIDS than females (AOR = 1.60; 95% CI = 1.21, 9.12).

**Conclusions:**

In this study, knowledge, attitudes, and practices of people with disabilities in relation to HIV/AIDS and other STIs were relatively low. This is clear evidence that HIV programs need to ensure that people with disabilities can access basic knowledge about HIV/AIDS and STIs.

## Introduction

Globally, more than one billion people (15% of the world’s population) live with a disability. Four out of five people with disabilities (PWDs) live in developing countries [1]. In Ethiopia, more than 15 million PWDs live with physical, sensory (hearing and visual loss), intellectual disabilities, or a combination of these disabilities [2]. The incidence of disabilities has been increasing due to accidents and the increase in chronic health conditions including HIV [3, 4].

Human immune virus (HIV) and other sexually transmitted infections (STIs) and disability are linked in several ways [5]. Although HIV-related data on PWDs are limited, growing evidence suggests PWDs are more likely to encounter factors that put them at higher risk of HIV and other STI infection than people without disabilities [6]. Persistent discrimination against and exclusion of PWDs increase their vulnerability of HIV infections [7]. Furthermore, there is a misconception that PWDs are sexually inactive and they are frequently overlooked in HIV prevention and AIDS (acquired immune deficiency syndrome) programs [3, 5]. They are highly vulnerable to sexual abuse and experience social marginalization, which places PWDs at increased risk of HIV and STI infection [7, 8].

Although people with disabilities require the same HIV/AIDS information, services and supports as all other members of society, they are excluded and neglected in all of the sectors responding to HIV and STI infections [3]. Remarkably, they are notably absent from equitable access and are often treated as a low priority for those services [8, 9]. This access is hindered by several factors such as stigma and discrimination based on their disability status and/or type of disability. Healthcare services are often not physically accessible and lack sign language interpreters and other information formats such as Braille, audio, or plain language which can provide advice on HIV-related issues including sexually transmitted disease [4, 10, 11]. As a result of these factors, PWDs have inefficient information about the prevention and transmission of STIs [12] and they were more likely to describe incorrect modes of HIV transmission and prevention [13].

There is a growing body of literature that recognizes a variety of issues that affect knowledge, attitudes, and practices in relation to HIV/AIDS and other STIs among people with disabilities. For example, a literature review from developing countries [10] and a research from Uganda [9] suggested that PWDs have very limited knowledge about HIV and other STIs because they are frequently excluded from access to HIV/AIDS and reproductive health information because of their disability status. Sociodemographic characteristics such as type of disability marital and educational level were also reported as potential predictors of knowledge and practices of PWDs in relation to HIV/AIDS because PWDs’ interest in seeking SRH-related services and information may be affected by these variables [5, 11].

HIV/AIDS is now the leading cause of morbidity and mortality among adults in Ethiopia. The overall prevalence of HIV in Ethiopia was found to be 0.9 and 72, 2248 of adults were infected with HIV in 2017 [14]. To best of the researchers’ knowledge, there is also no official statistics exist on HIV prevalence among PWDs, which creates a challenge for understanding the extent of the pandemic in this population. Moreover, lack of attention to HIV/AIDS among PWDs is prominent and continuing. People with disabilities in Ethiopia have limited access to sexual and reproductive health information including HIV/AIDS and other STIs [4] and have an increased risk of HIV/AIDS and other STIs-related problems. They often lack knowledge of HIV/AIDS and other STIs [4, 15]. Lack of access to sexual and reproductive health information, in turn, make PWDs highly vulnerable to different types of SRH-related problems like STI/HIV, unwanted pregnancy, or unsafe abortion [16, 17].

At present, however, little is known about the knowledge, attitudes and practices of sexually transmitted diseases including HIV/AIDS and other STIs, and there is inadequate data on the sexual and reproductive health needs of PWDs in Ethiopia. The aim of this study was, therefore, to identify which individual factors best predict knowledge, attitudes, and practices in relation to HIV/AIDS and other STIs among a sample of PWDs in North-shewa zone, Ethiopia. An examination of the knowledge, attitudes, and practices regarding sexually transmitted diseases such as HIV/AIDS and STIs are the cornerstones to combat these diseases. Besides, evidence-based data on the sexual and reproductive health needs of PWDs make their needs visible for policymaking and program designing to protect them from infections.

## Methods

### Study setting and period

A cross-sectional survey was conducted from June to October 2019 in the North-shewa zone, Amhara regional state, Ethiopia. The Debre Berhan town (the zone city) is located 130 kilometers far from Addis Ababa (the capital city of Ethiopia). The zone has 10 functional disability-support organizations: one is found in Debre Berhan town and nine are found in the other nine districts of the zone. The rest 15 districts have no functional disability support-organization. Overall, these disability-support organizations comprised a total number of 1500 PWDs. These organizations support all PWDs (physically handicapped, hearing loss, partial mental illness, visual loss, multiple impairments). These disabled people’s organization provide advocacy service, provide life skill training and support to live in the community, get involved in work (*unpublished zonal health department report*, *2019)*.

### Participants and sampling procedures

All the reproductive-age groups of PWDs who enrolled in the disability-support organizations in North-shewa zone were the study participants. Those who were critically ill due to medical problem at the time of study and unable to communicate and respond to questionnaires were excluded from the study. People with dual disabilities such as unable to see plus unable to hear were also excluded from the study.

The sample size was calculated using a single population proportion formula. The assumptions were: the proportion of PWDs who had reproductive health knowledge was 79.6% (p = 0.796) [16], with 95% confidence interval (CI) to be 1.96, and margin of error to be 5%. With the above assumptions, the single population formula yields 250. Considering a design effect of 1.5, (250×1.5 = 375) and adding a non-response rate of 10% (375+37 = 412), a total sample size of 412 respondents were selected.

Of ten disability-support organizations, five of them were selected by lottery method. The disability-support organizations have registry which contains lists of people with disabilities. Each registry contained, on average, 320 PWDs and about 120 females were registered in each organization. The registry contained contact addresses of the participants and it was used as the sampling frame in this study. Then, we employed probability proportional to size to recruit 412 participants. From each disabled people’s organization, every four study participants were selected from the list of the registry (using a systematic random sampling technique) which gave nearly equal number of respondents to each organization.

### Data collection

A pre-tested questionnaire was used to collect the data. The structured questionnaire was designed in English then translated into Amharic (native language) and back into English to ensure consistency. A revision was made for some of the questions after the pretest. The data were collected by trained enumerators through a face-to-face interview. Five data collectors and five supervisors participated in the study. One of the data collectors was a certified sign language interpreter who could collect data from participants who have a hearing loss. Data completeness were checked by the investigators and supervisors.

#### Measurements

Most of the questioners were taken from the 2016 Ethiopian demographic health survey [18] and from peer-reviewed literature [10, 15, 19–21]. The questions comprised the following sections; socio-demographic characteristics and questions which examined the respondents’ knowledge, attitudes, and practices of HIV/AIDS and other STIs.

Knowledge questions in relation to HIV/AIDS were: What are the ways of HIV transmission? What are the ways of HIV prevention? Respondents answered either *“Yes”* or “*No”* or *“Do not know”* from the listed options. Based on these questions, mean scores of knowledge about HIV/AIDS were calculated to classify the respondents into two groups (knowledgeable and not knowledgeable). Respondents who answered *“Yes”* were considered as correctly answered and those who answered *“No”* and *“I do not know”* were considered as not answered correctly. The mean score of knowledge about HIV/AIDS was computed by adding up the score of correct response from the listed options. Respondents who scored the mean and above the mean score of correctly answered questions were classified as knowledgeable, less than the mean score of correct answers were classified as not knowledgeable towards facts in relation to HIV/AIDS.

Knowledge questions about STIs were: what is STIs? Which type of STIs did you know? Which signs/symptoms of STIs did you know? Which are the prevention methods of STIs? What are the complications of untreated STIs? Then, respondents answered either *“Yes”* or “*No”* or *“Do not know”* from the listed options. Similarly, the mean scores of knowledge about STIs were calculated from the listed options to classify the respondents into two groups (knowledgeable and not knowledgeable). Respondents who scored the mean and above the mean score of the correctly answered questions were classified as knowledgeable, less than the mean score of correct answers were classified as not knowledgeable about STIs.

The attitudes of respondents towards facts in relation to HIV/AIDS and other STIs were assessed with eleven and eight attitudinal questions, respectively. All attitudinal statements were three-point Likert-scale items (1 = yes (agree), 2 = no (disagree), 3 = do not know) and stated positively. The respondents could choose one of the three possible response categories. The respondents’ attitude was computed by adding up the score of correct response form attitudinal statements. Respondents who scored the mean and above the mean score of the correctly answered questions were classified as having a good attitude towards facts in relation to HIV/AIDS and other STIs. Cronbach’s alpha was computed to determine the internal reliability of the attitudinal aspects of questions towards facts of HIV/AIDS and STIs. The reliability scores are presented in Table 1.

**Table 1:** Reliability score of attitudinal aspects of questions towards HIV/AIDS and STIs scale.

Scale	Cronbach’s alpha value
Attitudinal aspects of questions towards HIV/AIDS	0.765
Attitudinal aspects of questions towards STIs	0.819

The mean score of the respondents’ attitude towards facts of HIV/AIDS was computed from these eleven attitudinal questions, and the mean score of the respondents’ attitude towards STIs was computed from eight attitudinal questions. Respondents who scored the mean and above the mean score of attitudinal statements were considered as having a good attitude and less than the mean score as a poor attitude for both HIV/AIDS and STIs. Concerning HIV testing practice, it was computed by calculating the percentage of PWDs who had been ever tested for HIV infections.

### Data analysis

Data were checked for completeness and inconsistencies. Epi-data version 3.1 was used for data entry and data were exported to SPSS version 21. Descriptive statistics were computed. Logistic regression was performed to analyze the data. Those independent variables which were statistically significant in the bivariate model (*p-value < 0*.*05*) were entered into the multivariable analysis. In the final model, a significant association was declared at a p-value of less than 0.05. The results were presented in texts and tables with adjusted odds ratio (AOR) and the corresponding 95% CI.

### Ethical considerations

Ethical approval was obtained from the research and an ethical review committee of Debre Berhan University. Written informed consent was obtained from each respondent. All the information obtained from the study participants were kept confidential throughout the process of study, and the name of the respondent was replaced by code. Withdrawal from the study at any point if they wished was assured.

## Results

### Socio-demographic characteristics

A total of 397 respondents were interviewed with a response rate of 96.4%. The mean age of the respondents was 27.7(±7.1SD) years. Fifty-six percent of respondents were female and 48.1% of participants were single. The highest number of study participants were Orthodox Christians (93.0%). Regarding the educational level of the respondents, 48% of respondents completed primary education (Table 2).

**Table 2:** Sociodemographic characteristics of respondents in North-shewa zone, Ethiopia, 2019.

Socio-demographic characteristics (N = 397)	Categories	N (%)
Form of disability of respondents	Partial mental illness	13(3.3)
	Hearing loss	57(14.4)
	Visual loss	94(23.7)
	Impaired mobility	197(45.2)
	Multiple disabilities	53(13.3)
Age	18–30	271(68.1)
	31–40	47(11.9)
	41–50	79(20.0)
Sex	Female	173(43.7)
	Male	224(56.3)
Marital status	Married	142(35.9)
	Single	191(48.1)
	Divorced/widowed	64(15.9)
Religion	Orthodox	369(93.0)
	Muslim	19(4.8)
	Protestant/Catholic	9(2.3)
Educational status	No education	94(23.7)
	Primary education	194(48.9)
	Secondary education	52(13.0)
	College education	57(14.4)
Work status	Had no job	163(41.1)
	Student	63(15.9)
	Employed	57(14.4)
	Lottery seller	41(10.3)
	Others(beggar, tailor)	73(18.3)
Living condition	With parents	105(26.3)
	With relatives	21(5.2)
	With friends/peers	124(31.4)
	With partner	93(23.5)
	Alone	54(13.6)

### Knowledge and practice of respondents regarding HIV/AIDS

Ninety-eight percent of respondents had ever heard about HIV/AIDS whereas three-fourth of them had ever heard about VCT (voluntary counselling and testing). Forty-two percent of the respondents knew HIV transmissions and 57.1% of respondents knew HIV prevention methods. Remaining faithful to partner was the most reported ways of HIV prevention methods (90.5%). Overall, 47% of respondents were knowledgeable in relation to HIV/AIDS (95% CI 41.3, 54.6). Regarding practice of HIV testing, only 23% of respondents had been ever tested for HIV infection (95% CI 11.9, 34.8) (Table 3).

**Table 3:** The respondents’ knowledge of HIV/AIDS in North-shewa zone, Ethiopia, 2019.

Variables	Yes	No
Have you ever heard about HIV/AIDS?	389(98.1)	8(1.9)
**What are the ways of HIV transmission? (n = 389)**		
Unsafe sexual intercourse	368(94.7)	28(5.3)
Sharing needles and syringes	319(82.1)	78(17.8)
Blood transfusion	301(77.4)	96(22.6)
During pregnancy	260(66.9)	137(35.1
During childbirth	231(59.3)	166(40.7)
Through breast milk	181(46.4)	216(55.6)
Through mosquito and another insect bite	72(18.6)	325(81.4)
Casual contact with a person (handshaking…)	18(4.6)	379(95.4)
**Average knowledge of HIV transmissions**	**164(42.2)**	**233(57.8)**
**What are the ways of HIV prevention? (n = 389)**		
Abstain from sexual intercourse	286(73.5	111(26.5)
Use a condom during sexual intercourse	284(73.0	113(27.0
Remain faithful to a partner	352(90.5)	45(9.5)
Avoid contaminated sharp objects	228(58.6)	169(41.4)
Avoid unsafe injections	133(34.2	264(65.8)
Avoid sex with sex workers	154(39.5	243(60.5)
**Average knowledge of HIV preventions**	**222(57.1)**	**175(42.9)**
Have you ever heard about VCT?	294(75.7)	103(24.3)
Have you ever tested for HIV?	90(23.2)	307(76.8)
**Overall HIV knowledge level**	**Knowledgeable**	**Not knowledgeable**
	**184(47.3)**	**205(52.7)**

### Attitude of respondents towards HIV/AIDS

The respondents’ attitude towards facts of HIV/AIDS was computed from eleven attitudinal statements. Thus, 62.7% of the respondents were above the mean score and they were considered as having a good attitude towards evidence of HIV/AIDS (95% CI 54.9, 71.6) (Table 4).

**Table 4:** The respondents’ attitude towards HIV/AIDS in North-shewa zone, Ethiopia, 2019.

Variables (n = 389)	Agree	Disagree	Do not know
Do you think a person can get HIV for the first time he or she has sex?	256(65.9)	58(14.8)	75(19.3)
Do you think, by looking carefully, one can know if someone has HIV?	66(17.0)	248(63.7)	75(19.3)
Do you think HIV/AIDS is severed and more affects youth?	292(75.2	61(15.6)	36(9.3)
Do you think premarital sex for youths is not supported?	245(63.0)	98(25.2)	46(11.9)
Do you think AIDS patients should be isolated for the safety of others?	53(13.7)	291(74.8)	44(11.5)
Do you think discussing condoms or contraceptives with young people promote promiscuity?	96(24.8)	235(60.4)	58(14.8)
Do you think that using a condom is a sign of not trusting a partner?	168(43.3)	172(44.1)	49(12.6)
Wife has a right to refuse unprotected sex with her husband if she wants to use a condom and if a husband not?	225(57.8)	95(24.4)	69(17.8)
Do you think a person having multiple sex partners has a high risk of acquiring HIV?	316(81.1)	27(7.0)	46(11.9)
Do you think HIV infected person can live longer if she or he is taking antiretroviral treatment?	288(74.1)	62(15.9)	39(10.0)
Do you think eating healthy foods can keep a person from getting HIV?	79(20.4)	255(65.6)	55(14.0)
**The overall attitude of HIV/AIDS**	**Good attitude**	**Poor attitude**	
	**244(62.7)**	**145(37.3)**	

### Knowledge of participants about STIs

As indicated in Table 5, 93% of respondents had ever heard about STIs. The knowledge of STIs types and major signs/symptoms of STIs was 44.9% and 37.6%, respectively. Likewise, the respondents’ knowledge of STIs transmission and prevention mechanisms was 44.5% and 59.7%, respectively. Overall, 46% of respondents had inclusive knowledge about STIs (95% CI 39.6, 50.1) (Table 5).

**Table 5:** The respondents’ knowledge of STIs in North-shewa zone, Ethiopia, 2019.

Variables	Yes	No
Have you ever heard about STIs? (n = 337)	371(93.4)	26(6.6)
**What is STIs (n = 371)**		
Illnesses transmitted by sexual intercourse	320(86.2)	30(8.1)
Do not know	21(5.7)	
**Which type of STIs did you know? (n = 371)**		
Gonorrhea	279(75.2)	92(24.8)
Syphilis	265(71.4)	106(28.6)
Chancroid	216(58.3)	155(41.7)
Lymphogranuloma venerum	182(49.0)	189(51.0)
HIV/AIDS	234(87.3)	47(12.7)
Do not know	17(4.5)	
**Average knowledge of the type of STIs**	**167(44.9)**	
**Which signs/symptoms of STIs did you know? (n = 371)**		
Genital ulcer	284(76.5)	87(23.5)
Genital discharge	249(67.1)	122(32.8)
Pain during urination	262(70.6)	109(29.4)
Genital swelling	195(52.5)	176(47.5)
Do not know	73(19.7)	
**Average knowledge of signs/symptoms of STIs**	**139(37.6)**	
**Which risks of STIs transmission did you know? (n = 371)**		
Unprotected sex	328(88.5)	43(11.5)
Inconsistent condom use	259(69.7)	112(30.3)
Having multiple partners	270(72.8)	101(27.2)
Sex work	218(58.7)	153(41.3)
Pregnancy	79(21.3)	292(78.7)
Sharing cloths	84(22.7)	287(77.3)
Do not know	13(3.4)	
**Average knowledge of risks of STIs transmissions**	**165(44.5)**	
**Which prevention methods of STIs did you know? (n = 371)**		
Screening and diagnosis of risky individuals	204(54.9)	167(45.1)
Treatment of patients & their sexual partners	221(59.6)	150(40.4)
Use of condom prevent STIs	261(70.4)	110(29.6)
Do not know	32(8.7)	
**Average knowledge of prevention methods of STIs**	**222(59.7)**	
**What are the complications of untreated STIs? (n = 371)**		
Upper genital tract infections	208(56.1)	163(43.9)
Infertility	186(50.2)	185(49.8)
Cervical cancer	232(62.5)	139(37.5)
Enhanced transmission & acquisition of HIV	280(75.5)	91(24.5)
Others (bleeding,)	41(11.1)	
**Average knowledge of complication of untreated STIs**	177(47.7)	
**Overall knowledge of STIs**	**Knowledgeable**	**Not knowledgeable**
	**174(46.9)**	**197(53.1)**

### Attitude of participants towards STIs

As demonstrated in Table 6, the respondent’s attitude on STIs was computed by adding up the score of correct response form eight attitudinal statements. Accordingly, 96%respondents were above the mean score and they were considered as having a good attitude towards helpful facts and preventive methods of STIs (95% CI 62.9, 74.8) (Table 6).

**Table 6:** The respondents’ attitude towards STIs in North-shewa zone, Ethiopia, 2019.

Variables (n = 371)	Agree	Disagree	Do not know
Do you think STIs are not as dangerous as they can be cured?	104(28.1)	201(54.1)	66(17.8)
Do you think screening for STIs is good?	301(81.1)	27(7.4)	39(10.5)
Do you think a person who is infected with STI must be treated?	302(81.5)	54(14.4)	15(4.1)
Do you think isolating a person who is infected with STI can help prevent the spread of the disease?	100(27.0)	235(63.3)	36(9.6)
Do you think STIs affect the marital relationship?	238(64.1)	88(23.7)	45(12.2)
Do you think a person who is infected with STIs can be cured?	158(42.6)	146(39.3)	67(18.1)
I do not mind if others know that I am with STIs	183(49.3)	139(37.4)	49(13.3)
Do you think STIs patients pay the price for their immoral life?	83(22.4)	178(47.9)	110(29.7)
**Overall STIs attitude level**	**Good attitude**		**Poor attitude**
	**258(69.6)**		**113(30.4)**

### Determinants of knowledge about HIV/AIDS and other STIs

In the bivariate model, the sex of the respondents and previous information about VCT were statistically significant with knowledge of HIV/AIDS. In the same model, age and marital status of the respondents were also associated with their knowledge of STIs. Those variables which had a significant association in the bivariate model were entered into multiple logistic regression analyses. The results of the analysis showed that male respondents were 1.6 times more likely to have good knowledge of HIV/AIDS than females (AOR = 1.60; 95% CI = 1.21, 9.12). Likewise, married respondents were two times more likely to have good knowledge of STIs than single (AOR = 2.23; 95% CI = 1.92, 10.72) (Table 7).

**Table 7:** Determinants of knowledge about HIV/AIDS and other STIs among respondents in North-shewa zone, Ethiopia, 2019.

Variables associated with knowledge of HIV/AIDS		Not knowledgeable	Knowledgeable	COR(95% CI)	p-value	AOR(95% CI)	p-value
Sex of the respondents	Female	101(49.3)	68(37.0)	1		1	
	Male	104(50.7)	116(63.0)	1.66(1.21,7.32)	0.001	1.60(1.21, 9.12)	0.011
Ever heard about VCT	Yes	180(87.6)	118(64.0)	1		1	
	No	25(12.4)	66(36.0)	4.03(1.10,6.81)	0.010	3.17(0.89,7.26)	0.171
**Variables associated with knowledge of STIs**							
Age of the respondents	18–30	123(62.4)	93(53.2)	1		1	
	31–40	40(20.3)	42(23.4)	1.39(1.13, 9.77)	0.021	1.31(0.92,11.91)	0.502
	41–50	34(17.3)	39(23.4)	1.52(0.87, 5.77)	0.231	1.43(0.41, 6.17)	0.211
Marital status of the respondents	Single	82(41.6)	41(23.6)	1		1	
	Married	76(38.6)	92(52.9)	2.42(1.81,9.11)	0.006	2.23(1.92,10.72)	0.007
	Divorced/widowed	39(19.8)	41(23.5)	2.10(0.86,22.51)	0.171	2.21(0.86,23.17)	0.140

## Discussions

This study highlighted interesting insights into the knowledge, attitudes and practices of HIV and other STIs among PWDs in North-shewa zone, Ethiopia, unfortunately, there is inadequate data on the sexual and reproductive health status of people with disabilities. As underscored by the findings of this study, our results bring us how sexual health information and services are delivered among these population groups. In this study, nearly half of the respondents were knowledgeable in relation to HIV/AIDS and other STIs. However, this figure is lower than the study conducted in Ghana [17] and Hawassa city, Ethiopia [11]. The possible explanation could be the difference in access to healthcare services and information and the attention given to the sexual health of PWDs by the local health departments. Our finding suggest that there is indeed lack of sexual health information and services in the study area and thus SRH-related programs need to ensure PWDs can access basic knowledge about HIV/AIDS and other STIs.

Additionally, 37.6% of our study participants knew the major signs/symptoms of STIs which was slightly higher than a study conducted in Ifakara, Tanzania where 32.7% of study participants were able to mention only one sign/symptoms of STIs [22]. This could be noteworthy that PWDs have restricted access to sexual and reproductive health services including sexually transmitted infections, and they are often socially isolated in obtaining those services. It could also be explained that PWDs might have different interests in seeking STIs-related services and information.

As demonstrated in the results of this study, 62% of our respondents had favorable attitude towards facts of HIV/AIDS while 69% of respondents had good attitude towards helpful facts and preventive methods of STIs. This finding is consistent with previous research conducted in Hawassa city, Ethiopia [11] and in Zambia [23]. It is interesting to note that the attitude of participants may be influenced by the social determinants of HIV/AIDS and other STIs. Concerning the practice of HIV testing, only 23.2% of participants have ever been tested for HIV infections. In line with our finding, PWDs were less likely to be tested for HIV in Cameroon [3] and Uganda [24]. Similarly, as reported by previous research, families of PWDs may not encourage them to get HIV services [17, 23, 25]. The finding is more important in Ethiopia because stigma and social discrimination among PWDs exist in different aspects and social and cultural norms favour toward non-disabled people. Besides, these population groups are discouraged from HIV services as a result of negative conventions of health workers that they are not sexually active [5].

As demonstrated in the multivariate analysis, age, educational status, work status, living condition of the study participants did not have significant associations with the knowledge, attitudes and practices of HIV and other STIs. This indicates that knowledge, attitudes, and practices of PWDs in relation to HIV/AIDS and other STIs are not be affected by these variables. However, our result showed that married PWDs were two times more likely to have good knowledge of STIs than being single. In agreement with our finding, Mekonnen et al also reported that married PWDs were more knowledgeable about HIV/AIDS than unmarried [11]. The possible explanation could be that couples may be more concerned about HIV/AIDS and they often seek knowledge about sexual activity. Also, couples have a chance to discuss each other and share knowledge in relation to HIV/AIDS. Although females with disabilities are highly vulnerable to HIV infection and its effects, they are less likely to have HIV/AIDS-related knowledge [19]. This is also confirmed in our report in which male respondents were 1.6 times more knowledgeable about HIV/AIDS than females. This is probably because females with disabilities are often marginalized from community-based awareness activities of sexual and reproductive health including HIV and other STIs prevention. Besides, females with disabilities have a limited access to sexuality education than males with disabilities [26] and females with disabilities are discouraged from discussing HIV/AIDS and sexual-related issues in the study area due to some cultural influences.

Overall, our study adds to the remarkable body of evidence on knowledge, attitudes and practices of people with disabilities in relation to HIV/AIDS and STIs and has paramount public health importance to deliver appropriate SRH-information to people with disabilities. Our results also help policymakers to tailor suitable SRH-related programs that target the needs of PWDs in this context.

### Limitations of the study

Despite its strengths, this study has some limitations that must be acknowledged. Cross-sectional study design may not show the cause-effect relationship of variables and the results may be less reliable. Exploratory factor analysis was not conducted on the questionnaire. Furthermore, the study focused on disability-support organizations that may miss some PWDs in the community. The authors also acknowledge the possibility of information bias created by the sign language interpreters to interpret for respondents who had a hearing loss.

## Conclusions

In this study, knowledge, attitudes, and practices of people with disabilities in relation to HIV/AIDS and other STIs were relatively low. This is clear evidence that HIV programs need to ensure that people with disabilities can access basic knowledge about HIV/AIDS and STIs.

## Supporting information

S1 File English and Amharic language version of the questionnaire and consent form, 2019. (PDF)

## Abbreviations

AIDS: Acquired Immune Deficiency Syndrome
AOR: Adjusted Odds Ratio
CI: Confidence Interval
COR: Crude Odds Ratio
HIV: Human Immune Virus
SPSS: Statistical Package for the Social Sciences
PWDs: People With Disabilities
SRH: Sexual and Reproductive Health
STIs: Sexually Transmitted Infections
