# Supplementary material for: Determinants of knowledge, attitudes, and practices in relation to HIV/AIDS and other STIs among people with disabilities in North-Shewa zone, Ethiopia
Source: PLoS One. 2020 Oct 27;15(10):e0241312. doi: 10.1371/journal.pone.0241312 (PMC7591023; doi:10.1371/journal.pone.0241312)
Supplement: S1 File — (PDF) [file pone.0241312.s001.pdf]

English and Amharic language version of the questioner and consent form

Consent form

Dear Madam -----------------------------

My name is ------------ and currently working at Debre Berhan university. The aim of this study is assessing Knowledge, Attitude and Practice of person with disability towards reproductive health in North Showa Zone, Ethiopia. Findings from this research help health care provider to have information weather SRH service is disseminated or available to person with disability. This study will also enable to advise policy makers on strategies to minimize this group's problems making them vulnerable to different types of SRH-related problems like STI/HIV, unwanted pregnancy, or unsafe abortion.

Thus this interview is prepared for this purpose to get the right information on the topic. The information that will be obtained using this interview will be used only for research purpose. Confidentiality and anonymity is fully assured, as your name is not required and only the research team will have access to the results. It will not affect you in anyway.

Should you not take part in this study? If **yes,** continue to sign consent form

If No, stop here

I have been informed that the purpose of this study is assessing Knowledge, Attitude and Practice of person with disability towards reproductive health in North Showa zone, Ethiopia. I have understood that participation in this study is entirely voluntarily. I have been told that my answer to the question will not be given to anyone else and no reports of this study identify me in any way. I understood that participation in this study does not involve risks. I understood that Alemayehu Gonie is a contact person if I have question about the study or about my right as a study participant.

Respondent's Signature ___________Date___________

Start interview

Supervisor's name ________________ signature _________

Address of investigators: Tell: 0912379531 e-mail: alemayehugonie19@gmail.com

English language version of the questioner

Instruction to the interviewer: circle the number in front of the option based on the response.

Sr.	Part I: Demographic Characteristics	Options	Rema
1.1	Form of disability of respondents	1=Partial mental impairment 2=Hearing impairment<br>3=Visual impairment 4=Impaired mobility<br>5=Multiple impairment	
1.2	Age of respondents (in years)	----------------	
1.3	Sex of respondent	1=Female 2=Male	
1.4	Marital status of respondents	1= Married 2=Single 3=Separated 4= Widowed	
1.5	Religion	1= Orthodox 2= Muslim 3= Protestant<br>4=Others specify -------	
1.6	Educational status of respondent	1= Illiterate 2=Primary Education<br>3=Secondary Education 4= College Education	
1.7	Work status of respondents	--------	
1.8	Living situation of respondents	1=With parents 2=With relatives<br>3=With friends/peers 4=With partner 5=Alone<br>6=Orphanage 7= Others-----------------	
**Part II: Knowledge and practice assessment regarding STIs**			
4.1	Have you ever heard about STIs?	1=Yes 2=No **If no skip to Q 5.1**	
4.2	If **yes** for **Q 4.1,** what is/are STIs	1=Illnesses transmitted by sexual intercourse<br>2= Don't know	
4.3	Which type of STIs did you know?<br>(circle all possible answers)	1=Gonorrhea 2=Syphilis 3= Chanchroid<br>4= Lymphogranuloma venerum 5=HIV/AIDS<br>6=Others	
4.4	Did you know the main signs/symptoms of STIs?<br>(circle all possible answers)	1=Genital ulcer 2=Genital discharge<br>3=Pain during urination 4=Genital swelling<br>5= Don't know 6=Others	
4.5	Did you know the risks of STIs transmission?<br>(circle all possible answers)	1=Unprotected sex 2= Inconsistent condom use<br>3= Having multiple partners 4= Sex work<br>5= Pregnancy 6=Sharing cloths 7= others ------	
4.6	Did you know the prevention mechanisms of STIs?<br>(circle all possible answers)	1=Screening and diagnosis of risky individuals<br>2=Treatment of patients & their sexual partners	

		3= Use of condom prevent STIs 4=Others ------	
4.7	What are the complications of untreated STIs?<br>(circle all possible answers)	1= Upper genital tract infections 2=Infertility<br>3= Cervical cancer 4=Enhanced transmission &acquisition of HIV 5= others -------------	
4.8	Have you screened/seek screening for STIs in the past?	1=Yes 2=No	
4.9	If yes for Q 4.8, what was the result of your diagnosis?	1= negative /free from STI 2=Positive	
**Part III: Knowledge and practice assessment regarding HIV/AIDS**			
5.1	Have you ever heard about HIV?	1=Yes 2=No **If no skip to Q 5.4**	
5.2	If **yes for Q5.1,** what are the ways of HIV transmission?<br><br>(circle all possible answers)	1=Unsafe sexual intercourse<br>2=Sharing needles and syringes<br>3=Blood transfusion 4= During pregnancy<br>5= During child birth 6=Through breast milk<br>7= Through mosquito and other insect bite<br>8= Casual contact with a person (Hand shaking & sharing food…) 9=Others	
5.3	Did you know about ways of HIV prevention?<br>(circle all possible answers)	1=Abstain from sexual intercourse<br>2=Use condom during sexual intercourse<br>3=Remain faithful to a partner<br>4=Avoid contaminated sharp objects<br>5=Avoid unsafe injections<br>6=Avoid sex with sex workers 7=Others----	
5.4	Have you ever heard about VCT?	**1= yes 2= no**	
5.5	Have you ever tested for HIV?	1= yes 2= no	
5.6	If **no for Q** 5.5 what was your reason?	1=I didn't think it was important<br>2=Testing kit wasn't available<br>3=Partner refused 4=Other reasons-----------	
5.7	Do you have an intention to be tested for HIV?	1= yes 2= no	
**Part IV: Attitude assessment towards HIV(Do you agree or disagree with the statements about HIV (tick one)**			
6.1	A person can get HIV the first time he or she has sex.	1= yes(agree) 2=no(disagree) 3= don't know	
6.2	By looking carefully, one can know if someone has HIV.	1= yes(agree) 2=no(disagree) 3= don't know	
6.3	HIV/AIDS is sever and more affects youth	1= yes(agree) 2=no(disagree) 3= don't know	
6.4	Premarital sex for youths is not supported.	1= yes(agree) 2=no(disagree) 3= don't know	
6.5	AIDS patients should be isolated for the safety of others	1= yes(agree) 2=no(disagree) 3= don't know	

6.6	Discussing condom or contraceptive with young people promotes promiscuity.	1= yes(agree) 2=no(disagree) 3= don't know	
6.7	Using condom is a sign of not trusting partner.	1= yes(agree) 2=no(disagree) 3= don't know	
6.8	A wife has a right to refuse unprotected sex with her husband if she wants to use condom and if a husband not.	1= yes(agree) 2=no(disagree) 3= don't know	
6.9	A person having multiple sex partners has a high risk of acquiring HIV.	1= yes(agree) 2=no(disagree) 3= don't know	
6.10	HIV infected person can live longer if she or he is taking antiretroviral treatment (ART).	1= yes(agree) 2=no(disagree) 3= don't know	
6.11	Eating healthy foods can keep a person from getting HIV	1= yes(agree) 2=no(disagree) 3= don't know	
**Part V. Attitude assessment towards STIs(Do you agree or disagree with the statements about STIs (tick one)**			
7.1	Do you think STIs aren't dangerous as they can be cured?	1= yes(agree) 2=no(disagree) 3= don't know	
7.2	Screening for STIs is good	1= yes(agree) 2=no(disagree) 3= don't know	
7.3	Do you think a person who are infected with STI must get treatment?	1= yes(agree) 2=no(disagree) 3= don't know	
7.4	Do you think isolating a person who are infected with STI can help prevent spread of the disease?	1= yes(agree) 2=no(disagree) 3= don't know	
7.5	Do you think STIs are seriously affects the marital relationship?	1= yes(agree) 2=no(disagree) 3= don't know	
7.6	Do you think a person who are infected with STI can be cured from STI?	1= yes(agree) 2=no(disagree) 3= don't know	
7.7	I don't mind if others know that I am with STIs	1= yes(agree) 2=no(disagree) 3= don't know	
7.8	STIs patients pay the price for their immoral life	1= yes(agree) 2=no(disagree) 3= don't know	
**Thank you for your participation!!!!**			

Amharic version of the questioner

Instruction to the interviewer: circle the number in front of the option based on the response.

Sr.	Part I: Demographic Characteristics	Options	Remark
1.1	የአካል ጉዳቱ አይነት	1=የዓዕምሮ ህመም ያለባቸዉ 2=መስማት የተሳናቸዉ 3=ማየት የተሳናቸዉ 4= የአካል ጉዳት ያለባቸዉ 5=ሁለትና ከዛ በላይ የአካል ጉዳትያለባቸዉ	
1.2	የተሳታፊ እድሜ በዓመት	----------------	
1.3	የተሳታፊ ፆታ	1=ሴት 2=ወንድ	
1.4	የተሳታፊ የጋብቻ ሁኔታ	1= በለትዳር 2= ያላገባ/ች 3=የተፋቱ 4= የሞተባት/ችበት	
1.5	የተሳታፊ ሃይማኖት	1= ኦረቶዶክስ 2= ሙስሊም 3=ፕሮቴስታንት 4= ሌላ ካለ ይጠቀስ-----------------	
1.6	የተሳታፊ የትምህርት ሁኔታ	1= መደበኛ ት/ት ያልተማሩ 2=አንደኛ ደረጃ 3=ሁለተኛ ደረጃ 4=የኮሌጅ	
1.7	የተሳታፊ የሰራ ሁኔታ	------------------	
1.8	የተሳታፊ የኑሮ ሁኔታ	1= ከወላጆቹ ጋር 2=ከዘመድ ጋር 3=ከጋደኛ ጋር 4=ከትዳር ጓደኛ ጋር 5=ብቻየን 6=ከወላጅ አልባ ረጅ ድርጅት ጋር 7= ሌላ ካለ ይጠቀስ-----------------	
**Part IV: Knowledge and practice assessment regarding STIs**			
4.1	በግብረ-ስጋ ግንኙነት አማካኝነት የሚተላለፍ በሽታዎች ሲባል ሰምተሀ/ሽ ታዉቃለህ/ሽ?	1= አወን 2= የለም If **የለም, skip to Q 5.1**	
4.2	መልስዎ ለጥያቄ ቁጥር **4.1 አዎን** ከሆነ, በግብረ-ስጋ ግንኙነት አማካኝነት የሚተላለፍ በሽታ ምን ማለት ነዉ	1=በግብረ-ስጋ ግንኙነት የሚተላለፍ በሽታ ነው 2=አላዉቅም	
4.3	በግብረ-ስጋ ግንኙነት አማካኝነት ከሚተላለፍ በሽታዎች የትኞቹን ታዉቃለህ/ሽ? (circle all possible answers)	1=ጨብጥ 2=ቂጥኝ 3= ከርክር 4= ባምቡሌ 5=HIV/AIDS 6=ሌላ ካለ ይጠቀስ----------	
4.4	በግብረ-ስጋ ግንኙነት አማካኝነት የሚተላለፉ በሽታዎች ምን ምን ዋና ዋና ምልክቶች አላቸዉ (circle all possible answers)	1=የብልት አካባቢ መቁሰል 2=ከብልት የሚወጣ ፈሳሽ መኖር 3=ሽንት ሲሸኑ የማቃጠል ስሜት መኖር 4=የብልት አካባቢ እብጠት መኖር 5= አላቅም	

		6=ሌላ ካለ ይጠቀስ-----------------	
4.5	በግብረ-ስጋ ግንኙነት አማካኝነት ለሚተላለፉ በሽታዎች አጋላጭ የሆኑ የመተላለፊያ መንገዶች ምን ምን ናቸዉ?<br><br>(circle all possible answers)	1= ጥንቃቄ የጎደለዉ ግብረ-ስጋ ግንኙነት<br>2= በአገባቡና ሁልጊዜ ኮንዶም አለመጠቀም<br>3= ከብዙ ጓደኛ ጋር ፆታዊ ግንኙነት ማድረግ<br>4= በሴተኛ አዳሪነት መተዳደር/ 5= እርግዝና<br>6=ልብሶችን በጋራ በመጠቀም 7= ሌላ ካለ ይጠቀስ------	
4.6	በግብረ-ስጋ ግንኙነት አማካኝነት የሚተላለፉ በሽታዎችን መከላከያ መንገዶች ምን ምን ናቸዉ?<br>(circle all possible answers)	1=ተጋላጭ የሆኑ ሰዎችን ልየታ ማድረግ እና መመርመር<br>2=በበሽታዉ የተያዙ ሰዎችንና የፆታዊ ግንኙነት ጓደኛቸዉን ማከም 3= በአገባቡና ሁልጊዜ ኮንዶም መጠቀም 4=ሌላ ካለ ይጠቀስ-----------------	
4.7	በግብረ-ስጋ ግንኙነት አማካኝነት የሚተላለፉ በሽታዎች ካልታከሙ የሚያመጡት የጤና እክል ምን ሊሆን ይችላል?<br>(circle all possible answers)	1= የላይኛዉ የመራቢያ አካል በሽታ 2=መካንነት<br>3= የማህፀን ጫፍ ካንሰር 4= የ HIV መተላለፍን እና መያዝን ማፋጠን 5= ሌላ ካለ ይጠቀስ------------	
4.8	ከዚህ በፊት በግበረ-ስጋ ግንኑነት የሚተላለፍ በሽታዎችን ለማወቅ ምርመራ አድርገኽ/ሽ ታዉቃለህ/ሽ?	1= አወን 2= የለም	
4.9	ለጥያቄ ቁጥር 4.8 መልስዎ አዎን ከሆነ, የምርመራ ዉጤቱ ምን ነበረ?	1= ከ STI ነፃ ነበርኩ 2=STI ነበረበኝ	
**Part V: Knowledge and practice assessment regarding HIV/AIDS**			
5.1	HIV ሲባል ሰምተህ/ሽ ታዉቃለህ/ሽ?	1= አወን 2= የለም **If no skip to Q 5.4**	
5.2	ለጥያቄ ቁጥር **5.1** መልስዎ አዎን ከሆነ, የHIV መተላለፊያ መንገዶች ምን ምን ናቸዉ?<br><br>(circle all possible answers)	1=ጥንቃቄ የጎደለዉ ግብረ-ስጋ ግንኙነት<br>2=ስለታምና ሹል ነገሮችን መጋራ መጠቀም<br>3=ደም ንክኪ/ልገሳ 4= በእርግዝና ጊዜ ከእናት ወደ ልጅ<br>5= በወሊድ ጊዜ ከእናት ወደ ልጅ 6=ጡት በማጥባት<br>7= በወባ ትንኝ ንክሻና በሌሎች ተባዮች<br>8= በሽታዉ ካለባቸዉ ስዎች ጋር በንክኪ (በሰላምታ፣ አብሮ በመብላትና በመጠጣት….)<br>9=ሌላ ካለ ይጠቀስ----------------	
5.3	የHIV መከላከያ መንገዶች ምን ምን ናቸዉ?<br>(circle all possible answers)	1=መታቀብ 2=ኮንዶም መጠቀም	

		3=አንድ ለአንድ መወሰን<br>4=ስለታምና ሹል ነገሮችን በጋራ አለመጠቀም<br>5=ንፅህናዉ ባለተጠበቀ መርፌ መወጋት<br>6=ከሴተኛ አዳሪዎች ጋር ግብረ-ስጋ ግንኙነት አለማድረግ<br>7=ሌላ ካለ ይጠቀስ----------	
5.4	በፈቃደኝነት ላይ የተመሰረተ የምክርና የምርመራ አገልገሎት(VCT) ሲባል ሰምተህ/ሽ ታዉቃለህ/ሽ?	1= አወን 2= የለም	
5.5	የHIV ምርመራ አድርገህ/ሽ ታዉቃልህ/ሽ?	1= አወን 2= የለም **If no skip to Q 5.7**	
5.6	ለጥያቄ ቁጥር **5.5** መልስዎ አዎን ከሆነ, ያልተመረመርክበት/ሽበት ምክንያት ምን ነበር?	1=አይጠቅምም ብዬ ስላሰብኩ<br>2=የመመርመሪያ መሳሪያ/ kit/ ስለሌለ<br>3=የትዳር አጋሬ መመርመር ስላልፈለገ<br>4=ሌላ ምክንያት ካለ ይጠቀስ----------------	
5.7	ወደፊት HIV የመመርመር ፍላጎቱ አለህ/ሽ?	1= አወን 2= የለም	
**Part VI: Attitude assessment towards HIV(Do you agree or disagree with the statements about HIV (tick one)**			
6.1	አንድ ሰዉ ለመጀመሪያ ጊዜ የግብረ-ስጋ ግንኙነት ቢያደርግ HIV ሊይዘዉ ይችላል።	1= አዎ(እስማማለሁ) 2=የለም(አልስማማም) 3= አላቅም	
6.2	በጥንቃቄ በማየት አንድን ሰዉ HIV እደያዘዉ ማወቅ ይቻላል	1= አዎ(እስማማለሁ) 2=የለም(አልስማማም) 3= አላቅም	
6.3	ወጣቶች በHIV/AIDS በይበልጥና በከፋ ሁኔታ ይጠቃል።	1= አዎ(እስማማለሁ) 2=የለም(አልስማማም) 3= አላቅም	
6.4	ወጣቶች ከጋብቻ በፊት የግብረ-ስጋ ግንኙነት ማድረግ የለባቸዉም።	1= አዎ(እስማማለሁ) 2=የለም(አልስማማም) 3= አላቅም	
6.5	ለሌሎች ሰዎች ደህንነት ሲባል የAIDS ህመምተኞች ከሌሎች ሰዎች መነጠል አለባቸዉ።	1= አዎ(እስማማለሁ) 2=የለም(አልስማማም) 3= አላቅም	
6.6	ወጣቶችን ስለኮንዶም ማወያየት ሴሰኛ እዲሆኑ ያደርጋቸዋል።	1= አዎ(እስማማለሁ) 2=የለም(አልስማማም) 3= አላቅም	
6.7	ኮንዶም መጠቀም ጋደኛን ያለማመን ምልክት ነዉ።	1= አዎ(እስማማለሁ) 2=የለም(አልስማማም) 3= አላቅም	
6.8	ሚስት በኮነደም መጠቀም ከፈለገች ባል ባይፈልግም ጥንቃቄ የጎደለዉን የግብረ-ስጋ ግንኙነት የመከልከል መብት አላት።	1= አዎ(እስማማለሁ) 2=የለም(አልስማማም) 3= አላቅም	

6.9	ብዙ የፆታዊ ጋደኛ ያላቸዉ ሰዎች በ HIV የመያዝ እድላቸዉ ከፍተኛ ነዉ፡፡	1= አዎ(እስማማለሁ) 2=የለም(አልስማማም) 3= አላቅም	
6.10	በHIV የተያዙ ሰዎች የፀረ-ኤች.አይ.ቪ መድሃኒቶችን ከወሰዱ ለረጅም ጊዜ መኖር ይችላሉ፡፡	1= አዎ(እስማማለሁ) 2=የለም(አልስማማም) 3= አላቅም	
6.11	ጤናማ የሚባሉ ምግቦችን መመገብ በHIV እዳንያዝ ያደርጋል፡፡	1= አዎ(እስማማለሁ) 2=የለም(አልስማማም) 3= አላቅም	
**Part VII: Attitude assessment towards STIs(Do you agree or disagree with the statements about STIs (tick one)**			
7.1	በግብረ-ስጋ ግንኙነት አማካኝነት የሚተላለፉ በሽታዎች መዳን ስለሚችሉ አደገኛ አደሉም፡፡	1= አዎ(እስማማለሁ) 2=የለም(አልስማማም) 3= አላቅም	
7.2	በግብረ-ስጋ ግንኙነት አማካኝነት ለሚተላለፉ በሽታዎች ምርመራ ማድረግ በጣም አስፈላጊ ነዉ፡፡	1= አዎ(እስማማለሁ) 2=የለም(አልስማማም) 3= አላቅም	
7.3	በግብረ-ስጋ ግንኙነት አማካኝነት በሚተላለፉ በሽታ የያዘዉ ሰዉ የግዴታ መታከም አለበት፡፡	1= አዎ(እስማማለሁ) 2=የለም(አልስማማም) 3= አላቅም	
7.4	በግብረ-ስጋ ግንኙነት አማካኝነት በሚተላለፉ በሽታ የተያዘ ሰዉ በሽታዉን ወደሌሎች አዳያስተላልፉ ከሰዎች መነጠል አለበት፡፡	1= አዎ(እስማማለሁ) 2=የለም(አልስማማም) 3= አላቅም	
7.5	በግብረ-ስጋ ግንኙነት አማካኝነት በሚተላለፉ በሽታ መያዝ የጋብቻ ግንኑነትን/ትዳርን በእጅጉ ያዉካል፡፡	1= አዎ(እስማማለሁ) 2=የለም(አልስማማም) 3= አላቅም	
7.6	በግብረ-ስጋ ግንኙነት አማካኝነት በሚተላለፉ በሽታ የተያዘ ሰዉ መዳን ይችላል፡፡	1= አዎ(እስማማለሁ) 2=የለም(አልስማማም) 3= አላቅም	
7.7	በግብረ-ስጋ ግንኙነት አማካኝነት በሚተላለፉ በሽታዎች መያዜን ሌላ ሰዉ ቢያዉቅብኝ ግድየለኝም፡፡	1= አዎ(እስማማለሁ) 2=የለም(አልስማማም) 3= አላቅም	
7.8	በግብረ-ስጋ ግንኙነት አማካኝነት በሚተላለፉ በሽታዎች የታመሙ ሰዎች ለሰሩት ኢ-ሞራል(ግብረ ገብነት የጎደለዉ ስራ ስለሰሩ) ስራ ዋጋ እየከፈሉ ነዉ፡፡	1= አዎ(እስማማለሁ) 2=የለም(አልስማማም) 3= አላቅም	

በጥናቱ ስለተሳተፉ አመሰግናለሁ!!!!
